# Eosinophilic esophageal myositis: a rare cause of dysphagia

**DOI:** 10.1055/a-2409-0243

**Published:** 2024-09-19

**Authors:** Li Wang, Guo-Dong Yang, Ke Pu, Li-Ping Tao, Xian-Fei Wang, Xue-Mei Lin, Cong Yuan

**Affiliations:** 1 Department of Pathology, Institute of Basic Medicine and Forensic Medicine, North Sichuan Medical College, Nanchong, China; 2 Department of Pathology, Affiliated Hospital of North Sichuan Medical College, Nanchong, China; 3 Department of Gastroenterology, Affiliated Hospital of North Sichuan Medical College, Nanchong, China; 4 Digestive Endoscopy Center, Affiliated Hospital of North Sichuan Medical College, Nanchong, China; 5 117913 Department of Gastroenterology, Affiliated Hospital of North Sichuan Medical College, Nanchong, China

A 48-year-old man complained of intermittent dysphagia to solids for 2 months. A preliminary esophagogastroduodenoscopy indicated a normal appearance of esophageal mucosa and chronic atrophic gastritis ( Fig. 1 **a** ). An esophagram was unremarkable ( Fig. 1 **b** ). The patient declined esophageal high resolution manometry. He attempted treatment with esomeprazole for 1 month, but the dysphagia did not improve.

**Fig. 1:**
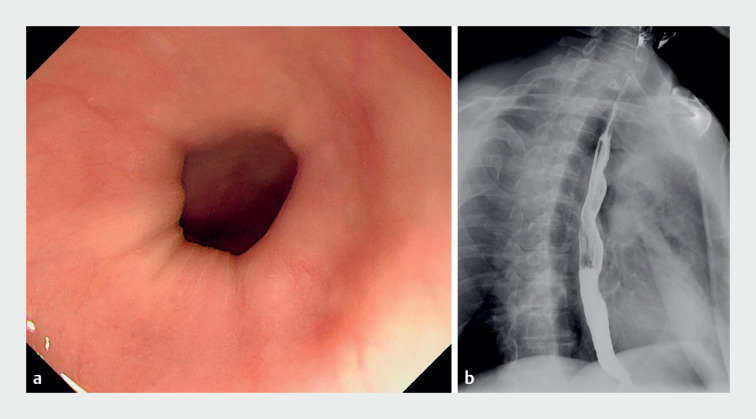
Preliminary images of the esophagus. **a** Esophagogastroduodenoscopy revealed a normal appearance of the esophageal mucosa. **b** Esophagram showed unremarkable findings.

Computed tomography scans were then performed and revealed circumferential thickening of the middle and distal esophageal wall, and no thoracic lump outside of the esophagus ( Fig. 2 **a** , Video 1 ). Endoscopic ultrasonography (EUS) demonstrated a gradual thickening of muscularis propria from the middle to lower esophagus, with muscularis propria layer thickness of 5.1 mm in the lower segment ( Fig. 2 **b** ). Six deep biopsy specimens were obtained from the lower esophagus. Hematoxylin and eosin staining demonstrated infiltration of more than 30 eosinophils per high power field (hpf) in the muscularis propria and no more than 5 eosinophils per hpf in the esophageal epithelium ( Fig. 3 ). Histologic and EUS findings strongly pointed to eosinophilic esophageal myositis (EoEM).

**Fig. 2:**
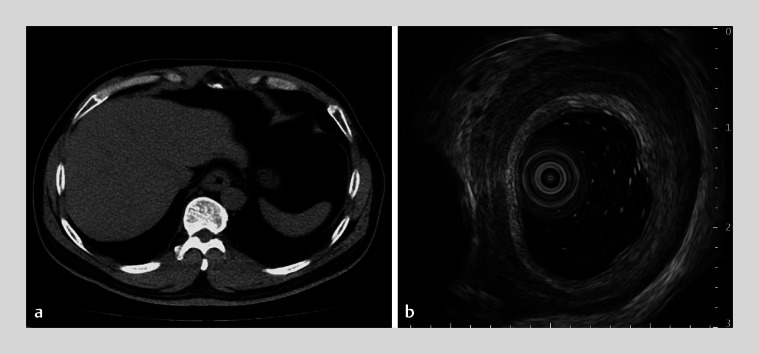
Computed tomography and endosonographic findings of the esophagus. **a** Computed tomography demonstrated circumferential thickening of the middle and distal esophageal wall. **b** Endoscopic ultrasonography revealed a gradual thickening of muscularis propria from the middle to lower esophagus, with muscularis propria layer thickness of 5.1 mm in the lower segment.

**Fig. 3:**
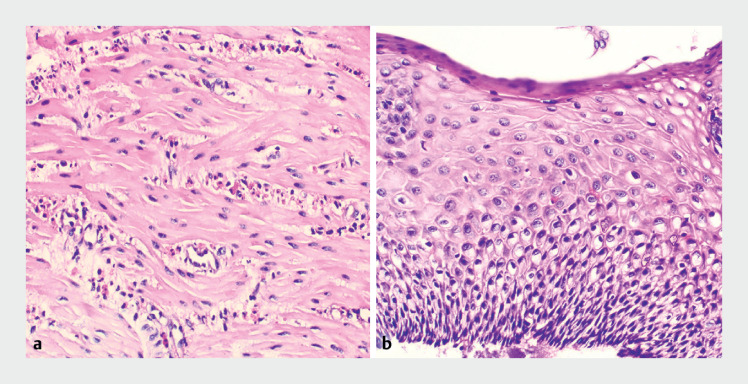
Histopathologic findings of the esophagus: **a** more than 30 eosinophils per high power field (hpf) infiltration in the muscularis propria; **b** no more than 5 eosinophils per hpf in the esophageal epithelium (hematoxylin and eosin staining, ×200).

Esophagogastroduodenoscopy showed a normal appearance of the esophageal mucosa. Computed tomography scans suggested esophageal wall thickening, which was seen on endoscopic ultrasonography as resulting from a thickened muscularis propria. Eosinophilic esophageal myositis was diagnosed.Video 1

Dysphagia improved after 1 month of swallowed topical corticosteroids (budesonide, 1 mg b.i.d) and is currently being maintained (budesonide, 0.25 mg b.i.d).

EoEM is an uncommon condition that is challenging for endoscopists. The symptoms of EoEM are not specific and the endoscopic findings are not typical, which may lead to a misdiagnosis or a delayed diagnosis. EoEM cases are usually diagnosed by EUS-guided fine-needle aspiration or muscle biopsies under peroral endoscopic myotomy 1 2 3 4 . In our case, we obtained tissue specimens simply by deep biopsy. In addition, EoEM causes thickening of the muscularis propria layer in the middle and lower esophagus 1 2 3 4 , rather than in the upper segment, which seems to be related to the abundance of smooth muscle in the middle and lower esophagus. More cases are needed to clarify this potential mechanism.

Endoscopy_UCTN_Code_CCL_1AB_2AC_3AH

## Notes

Endoscopy E-Videos https://eref.thieme.de/e-videos
*E-Videos* is an open access online section of the journal *Endoscopy* , reporting on interesting cases and new techniques in gastroenterological endoscopy. All papers include a high-quality video and are published with a Creative Commons CC-BY license. Endoscopy E-Videos qualify for HINARI discounts and waivers and eligibility is automatically checked during the submission process. We grant 100% waivers to articles whose corresponding authors are based in Group A countries and 50% waivers to those who are based in Group B countries as classified by Research4Life (see: https://www.research4life.org/access/eligibility/ ). This section has its own submission website at https://mc.manuscriptcentral.com/e-videos .

## References

[1] Sato H Takeuchi M Takahashi K Eosinophilic infiltration of the muscularis propria in a patient with jackhammer esophagus treated with per-oral endoscopic myotomy Clin Gastroenterol Hepatol 2015 13 e33 e34 25460551 10.1016/j.cgh.2014.11.005

[2] Yang X Xiang X Liu S A rare cause of dysphagia: diagnosis and treatment Gastroenterology 2022 163 e28 e30 10.1053/j.gastro.2022.07.020 36017786

[3] Tang Y Xiong W Yu T Eosinophilic esophageal myositis a plausible cause of histological changes of primary jackhammer esophagus: a case report Am J Gastroenterol 2018 113 150 152 10.1038/ajg.2017.433 29311727

[4] Igarashi R Irisawa A Shibukawa G Eosinophilic esophageal myositis diagnosed by endoscopic ultrasound-guided fine-needle aspiration biopsy: a case report Clin J Gastroenterol 2016 9 285 288 10.1007/s12328-016-0678-z 27503258

